# Effects of adoption of ecological farming practices on farm income in rural households: Evidence from Central Kenya

**DOI:** 10.1016/j.heliyon.2024.e34610

**Published:** 2024-07-14

**Authors:** Emma Wangari Kamau, Raphael Gitau, Hillary K. Bett

**Affiliations:** a Department of Agricultural Economics and Agribusiness Management Egerton University, Kenya, P.O. Box 412-20200, Kericho, Kenya; b Department of Agricultural Economics and Agribusiness Management Egerton University, Kenya, P.0. Box 536, 20115, Egerton-Njoro, Kenya

**Keywords:** Ecological farming, MESR model, Farm income, Sustainable farming, Kenya

## Abstract

Farmers have adopted ecological farming to overcome the adverse effects of climate change and conventional agriculture. However, the performance of ecological approaches, especially the economic outcomes of ecological practices adopted as a package, has yet to be widely documented in Kenya. This paper aims to fill this gap and generate pertinent information on the implications of adopting different ecological practices bundles on farm income. The study considers five ecological practices: crop diversification, composting, mulching, minimum tillage, and integrated pest management (IPM). This study employed a multinomial endogenous switching regression (MESR) model to capture the effect of adopting different combinations of ecological practices on farm income. The results reveal that 15.3 % of the surveyed households adopted a bundle combining composting, crop diversification, and IPM. Households that adopted a comprehensive package (comprising all five ecological practices) had the largest positive impact on farm income, increasing it by 9.2 %. This package replaces chemical inputs with locally available resources, restores degraded soils and diversifies production risks to increase food production and farm income. Farming experience, off-farm activities, farm size, perceptions of the effects of conventional agriculture, and drought and floods significantly influenced the adoption of the comprehensive package. This study’s findings imply that policymakers and related stakeholders should provide timely weather-related information, enforce sustainable land use laws, and establish targeted extension services and information campaigns to heighten the adoption of all five ecological practices and enhance household welfare by increasing farm income.

## Highlights

• The performance of ecological farming is not widely documented in Kenya.
• Effect of adoption of ecological farming practices modelled using MESR.
• Farm income varied with the bundles of ecological practices a household adopted.
• Households that adopted a comprehensive package increased farm income by 9.2 %.

## 1 Introduction

The conventional agricultural model has dominated the farming landscape over the years due to its ability to improve agricultural production. However, the continuous use of conventional farming practices resulted in widespread environmental degradation, biodiversity losses, high greenhouse gas (GHG) emissions, persistent hunger, diet-related diseases, broadened social inequalities among actors, and livelihood stresses for farmers around the world [[1], [2], [3]]. As a result, achieving food security has become a greater concern, particularly for smallholder farmers whose livelihoods depend on the availability of natural resources. Additionally, balancing food production, environmental conservation, and social inclusion is an immense future challenge.

In Africa, agricultural production is mainly characterised by smallholder, resource-poor farmers who often face the challenges of degraded natural resources, erratic and unpredictable rainfall, and expensive chemical fertilisers and pesticides. In these situations, farmers are better off with innovations that use locally available inputs and are compatible with specific ecological conditions. Alternative agricultural models of producing food in a sustainable manner, such as ecological farming, are gaining recognition among the farming communities. The ecological farming system integrates local knowledge into food production and provides a better relationship between agriculture and the environment and between the food system and society. In this study, ecological farming is conceptualized as a system of producing food that forgoes the use of agrochemical inputs and instead relies on ecological systems, biodiversity, and cycles adapted to local living conditions, ensuring the whole system is economically viable, socially just, and environmentally friendly [[4], [5], [6]]. Embracing ecological farming implies that farming households would rely on the use of compost, animal and farmyard manure, intercropping, crop rotation, crop and livestock integration, cover crops and mulching, mixed cropping, agroforestry, minimum or zero tillage, ridging, bush fallows, and integrated pest management on their farms [[7], [8], [9], [10]].

This paper considers different combinations of five ecological practices. The first ecological practice considered is crop diversification. It is a sustainable farming strategy that promotes crop diversity through multiple cropping, intercropping, planting regionally adapted varieties, and crop rotation to address population growth, climate change, and changing consumer habits [11,12]. The second ecological practice is mulching. Mulching helps farmers mitigate drought, water loss, and soil erosion, enhancing crop production, soil health, and biodiversity [[13], [14], [15]]. The third ecological practice considered is IPM. It is a science-based method that uses biological, cultural, physical, and chemical techniques to manage pest risks, preserve beneficial organisms, and foster resilient ecosystems [16,17]. The fourth practice is composting. It is a less-costly alternative to chemical fertilizers for resource-constrained farmers that improves soil fertility and crop production by reusing local resources. Compost manure increases organic content, water-holding capacity, and the macro and micronutrients available for plant uptake, improving plant growth and increasing crop productivity [18]. The fifth ecological practice is minimum tillage. It is a practice that conserves the soil by reducing soil disturbances and increasing water use efficiency [19]. Farmers have used ecological farming techniques over the years; however, in today’s agriculture, they are used in ways to provide sufficient food for a growing world population while conserving biodiversity and the ecosystem services on which agriculture depends and ensuring social justice and economic viability for farmers [20]. Adopting ecological practices has an impact on a household’s welfare. Evidence from the literature has shown that the adoption of a combination of sustainable agricultural practices (SAPs) has much greater benefits for welfare compared to the adoption of a single practice [[21], [22], [23], [24], [25]].

Agricultural production in Kiambu County has been declining mostly due to land degradation, poor access to agricultural inputs, crop and livestock diseases, and climate change. Farmers are addressing the challenges above by adopting conservation agriculture, climate-smart agriculture, and sustainable land management practices [26]. Some of the practices mostly incorporated by farmers in Kiambu County include cover cropping, intercropping, leguminous cropping, minimum tillage, crop rotation, mulching, organic manure use, integrated crop-livestock systems, agroforestry, biopesticides, and land terracing. Empirical evidence regarding the welfare effects of adopting individual ecological practices in Kenya such as minimum or zero tillage [27,28], IPM [29,30], organic fertilizer/manure [31], crop diversification [32,33], and agroforestry [34] exist, however, there is limited research work in Kenya that investigate farmers’ adoption of different combinations of ecological practices and their subsequent impacts.

This paper contributes to the existing literature on the relationship between eco-system-based practices and farm income in Kenya. Specifically, the paper evaluates the determinants of adopting different packages of ecological practices and the implications of their adoption on farm income, using Kiambu County as a case. Understanding the implications of the adopting different packages of ecological practices on farm income is important for designing effective financing and extension strategies that could promote adoption of the packages with the greatest positive effect on farm income. Further, the existing studies estimating the impacts of SAPs have considered risk reduction strategies like crop diversification and soil and water conservation technologies such as minimum tillage, crop residue retention, and the use of animal manure [[21], [22], [23], [24], [25]]. However, they have not given much attention to pest and disease management strategies. This study extends the existing literature by including IPM as one of the farming practices. The rest of the paper is structured as follows: Section 2 presents our empirical approach. In section 4, we provide the discussion of the results. The final section presents conclusion and policy implications.

## 2 Material and methods

### 2.1 Study area and sampling procedure

**Fig. 1:**
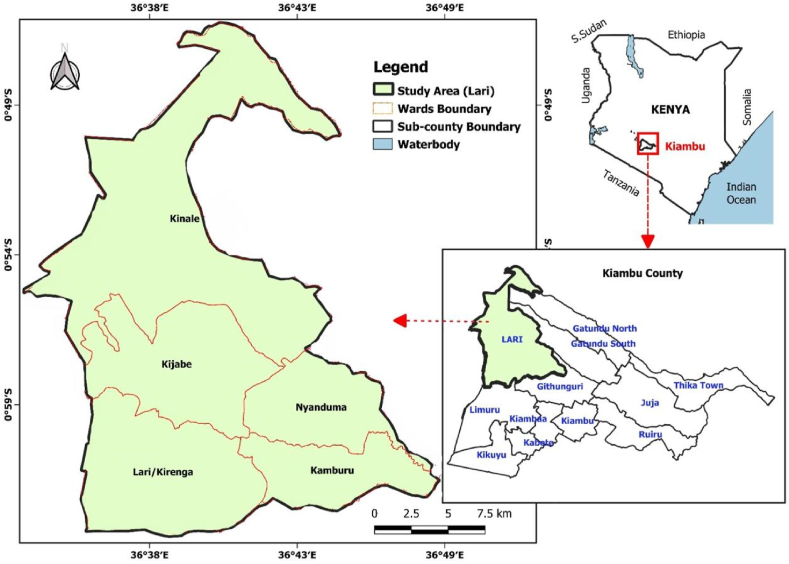
ArcGIS generated map showing the study area.

The data used in the study were collected in two wards in the Lari sub-county where ecological approaches are promoted by different institutions, namely, the Kamburu ward and the Lari/Kirenga ward. The map of the study area is shown in Fig. 1. A multi-stage sampling procedure was used to select respondents, whereby in stage one, Lari was purposively selected for the study because agriculture is the predominant economic activity; also, many institutions are promoting the adoption of ecological farming in the sub-county. The second stage involved purposively selecting two wards based on the many interventions received from different organisations. In the third stage, villages were randomly selected. Organisations that support agroecological practices in the region provided a list of farmers. There were 320 households that were selected through a systematic random sampling procedure.

### 2.2 Econometric model

To achieve the objective of this study, we used the multinomial endogenous switching regression model (MESR). We followed the studies by Refs. [25,35]. The uptake of ecological farming practices is non-random, and self-selection might occur. We treat adopting ecological farming practices as an endogenous variable because some unobservable factors, such as the farmer's preferences, management skills, motivation, and innate ability, which influence the farm household's decision to take up ecological farming practices or not, could also influence farm income. Unobserved factors are heterogeneous across farming households [36]. One method commonly used, mainly where self-selection bias occurs, is the Propensity Score Matching (PSM) method. However, the PSM approach only accounts for observed factors [37]. Thereby, employing MESR helps overcome the problems of selection bias and endogeneity brought about by observed and unobserved heterogeneity [38]. For instance, MESR estimates in this study provide insights into how the endogeneity and switching behaviours affect the relationship between the set of explanatory variables for specific technology choices and farm income. The MESR model is estimated in two stages:

In the first stage, this study employed the multinomial regression model to determine the factors influencing households' decisions to adopt ecological farming packages. In this case, the unordered responses have more than two outcomes; hence, a multinomial logit or probit model can be used. The multinomial probit model (MNP) has some practical limitations since it requires the evaluation of multiple integrals of the normal distribution and makes maximum likelihood infeasible for more than about five alternatives [39,40]. Thus, a multinomial logit model (MNL) was used. The MNL was used to capture the impact of independent variables on the likelihood of being in any of the identified categories relative to the reference category. Given the five ecological practices considered by the study, this study ended up with seven bundles for empirical estimations.

In the case of this study, we assume that households adopt a combination of those practices that help them maximise their revenues. Thus, for a farmer household i to choose a combination of practices, n, over any other alternative combination, m, can be illustrated as m ≠ n. This illustration shows that combination n provides higher expected revenues than any other alternative. It is a latent variable representing a household's expected revenue from adopting a set of ecological farming practices, n. As shown in equation (1), the latent variable is determined by observed household, socio-economic, and plot characteristics (Xi) and unobserved characteristics [40].

(1) $$U_{in}^{*}=X_{i}\beta_{in}+\varepsilon_{in}$$

where $X_{i}$ is observed exogenous variables (household, plot, and socio-economic characteristics) and $\varepsilon_{in}$ is unobserved characteristics. Assuming the error terms are identically and independently Gumbel distributed, the probability that a household, i, takes up a combination of practices, n, can be estimated using a multinomial logit regression model [41]

(2) $$P_{in}=\mathrm{Pr}\left(\;\varepsilon_{in}<0|X_{i}\right)=\frac{\exp\left(X_{i}\beta_{n}\right)}{\sum_{m=1}^{n}\;\exp\left(X_{i}\beta_{m}\right)}$$

Since MNL model (equation (2)) estimates provide only direction effects, this study calculated the marginal effects (dy/dx) to quantify the impact of each independent variable on the predicted probabilities. The marginal effects offer valuable information that is important in the decision-making process.

In the second stage, ESR was used to estimate the effect of adopting a package of ecological practices on farm income. We devised the outcome equation (farm income) conditional on whether a farm household takes up ecological farming. The base category was indicated as n = 1 (non-adoption). At least one ecological practice was used for the remaining set of practices (n = 2 … k). The farm income equation for each possible regime,n, is as shown by equation (3):

$$\text{Regime}\;1:Y_{1i}=\alpha_{1}Z_{i}+\mu_{1i}\;\text{if}\;i=1$$

(3) $$\text{Regime}\;n:Y_{ni}=\alpha_{n}Z_{i}+\mu_{in}\;\text{if}\;i=n$$

$Y_{i}$ represents the farm income for the ith farmer household; $Z_{i}$ is a vector of exogenous variables; $\alpha_{1}$ and $\alpha_{n}$ are parameters to be estimated; $\mu_{1i}$ and $\mu_{in}$ are the error terms.

The farm income of the ith household ($Y_{i}$) is observed if only one of the possible combinations of ecological farming practices is used. Some unobservable factors influencing the farm household's decision to adopt ecological farming practices could also influence farm income. Thus, the error term of the decision and outcome equations are non-zero, and ordinary least squares (OLS) estimates will not be consistent [42]. Endogenous switching regression (ESR) corrects the problem of unobserved selection bias using the inverse Mill's ratios/selectivity term of the choices [25,35]. The selectivity term is as expressed in equation (4):

(4) $$\lambda_{n}=\sum_{m\neq n}^{n}\rho_{n}\left[\frac{{\hat{P}}_{im}\;\ln\left({\hat{P}}_{im}\right)}{1-{\hat{P}}_{im}}+\ln\left({\hat{P}}_{in}\right)\right]$$

where ρ is the correlation coefficient of $\varepsilon_{in}$ and $\mu_{in}$. In the multinomial choice setting, there are n-1 selectivity terms, one for each alternative set of practices [25]. The inverse Mills ratio is then added into the aforementioned linear outcome equation (3) to yield equation (5);

$$\text{Regime}\;1:Y_{1i}=\alpha_{1}Z_{i}+\sigma_{1}\lambda_{1}+\upsilon_{1i}\;\text{if}\;i=1$$

(5) $$\text{Regime}\;n:Y_{ni}=\alpha_{n}Z_{i}+\sigma_{n}\lambda_{n}+\upsilon_{ni}\;\text{if}\;i=n$$

where; σ is the covariance between $\varepsilon_{in}$ and $\mu_{in}$, and $\upsilon_{ni}$ is the error term with an expected value of zero.

#### 2.2.1 Estimation of average treatment effects

The farm income of the ith household (Y) is observed if only combinations of one of the possible ecological farming practices are used. Some unobservable factors influencing the farm household's decision to adopt ecological farming practices could also influence farm income. Thus, the error term of the decision equation and the outcome equation are non-zero, and ordinary least squares (OLS) estimates will not be consistent [42]. Following [43], we calculate the predicted outcomes for adopters under observed conditions and counterfactual conditions (if adopters do not adopt):

Farm income for farming households that adopts ecological farming practices as observed in the sample

$$E\left(Y_{i2}|i=2\right)=Z_{i}\alpha_{2}+\sigma_{2}\lambda_{2}$$

(6) $$E\left(Y_{in}|i=n\right)=Z_{i}\alpha_{n}+\sigma_{n}\lambda_{n}$$

Farm income for farming households had they decided not to adopt ecological farming practices (counterfactual)

$$E\left(Y_{i1}|i=2\right)=Z_{i}\alpha_{1}+\sigma_{1}\lambda_{2}$$

(7) $$E\left(Y_{i1}|i=n\right)=Z_{i}\alpha_{1}+\sigma_{1}\lambda_{n}$$

The average treatment effect on treated (ATT) is presented by equation (8), which is the difference between the expected values of the outcome equations (6), (7). It represents the influence of adoption on farm income among farming household.

(8) $$E\left[Y_{i2}|i=2\right]-E\left[Y_{i1}|i=2\right]=Z_{i}\left(\alpha_{2}-\alpha_{1}\right)+\lambda_{2}\left(\sigma_{2}-\sigma_{1}\right)$$

### 2.3 Ethical consideration

The study adhered to the research ethics guidelines recommended by the Board of Postgraduate Studies at Egerton University. The study obtained informed consent from the interviewed farmers. It ensured that the principle of anonymity and voluntary participation was respected. The enumerators were trained to seek informed consent from the farmers and agree on voluntary participation in the study.

## 3 Results and discussion

### 3.1 Preliminary tests

All the fourteen independent variables used in the regression model were tested for multicollinearity. Variance inflation factor (VIF) was used to test for multicollinearity among the continuous explanatory variables. As a rule of thumb, a VIF value greater than the critical value of 10 indicates that multicollinearity is a major problem. The VIF values ranged from 1.06 to 2.06; the mean VIF was 1.31. Following this result, we concluded that multicollinearity was not a problem for our analysis. A pairwise correlation test was also used to test the linear relationship among the categorical explanatory variables used in the model. The coefficients were below the acceptable threshold of 0.5. This result confirms no strong correlation among the categorical explanatory variables. Further, the results from the Breusch-Pagan test reported a probability chi-square of 0.6054, which is greater than 0.05. Therefore, we did not reject the null hypothesis that the variance is constant among residues and concluded that heteroscedasticity is absent in the data.

### 3.2 Descriptive analysis

The descriptive statistics results for the variables used in the model are shown in Table 1. The results indicated that the average age of farmers was 50.23 years. The mean household size of the sampled farmers was 4.24. The mean number of years in school was 10.71. The mean number of years of farming experience was 19.72 years.

**Table 1:** Descriptive statistics for independent variables.

Variables	Description	Mean	SD
Age	Age of the household head (HH) in years	50.23	12.74
Gender	1 if HH is female and 0 otherwise	0.49	0.50
Household size	Number of people in a household	4.24	1.65
Education	Number of years of education of the HH	10.71	2.96
Off farm activities	1 if the HH is involved in other activities outside farming	0.38	0.49
Farming experience	Years of farming experience of the HH	19.72	11.94
Farm size	Size of land under cultivation in acres	1.04	1.34
Drought and floods	1 if household has experienced yield losses due to drought and floods and 0 if otherwise	0.63	0.48
Slope	1 if the slope of land under cultivation is very/fairly steep, 0 if otherwise	0.47	0.50
Soil erosion	1 if soil erosion on the cultivated land is very/moderately severe and 0 otherwise	0.15	0.36
Effect of Conventional agriculture	1 if the HH thinks the effects of using conventional agriculture are bad and 0 if otherwise	0.76	0.42
Membership	1 if the household belongs to a producer group and 0 if otherwise	0.52	0.50
Credit	1 if household access credit and 0 if otherwise	0.59	0.49
Kamburu ward	1 if household is in the Kamburu ward and 0 otherwise	0.64	0.48

Additionally, land available for farming was scarce in the study area due to other competing land-use activities. Farm size was an average of 1.04 acres. Male-headed households that adopted ecological packages accounted for 51 %, while female-headed households were 49 %. Moreover, farmers who participated in group activities accounted for 52 %. Belonging to a group enables farmers to learn from their peers and enhance their skills through farm demonstrations. Also, among the sampled households, 63 % stated they had experienced yield loss due to drought and floods. Most farmers (64 %) were located in the Kamburu ward.

Ecological farming practices are adopted in bundles of various combinations by households. Given the five ecological practices considered in this study, there were 31 possible ecological farming practices bundles, as shown in Table 2. Out of the possible 31 bundles, only 20 were adopted by the sampled households. Notably, those bundles with fewer observations, less than ten respondents, were dropped. To emphasize further, the bundle combining mulching and compost manure still had few observations (13 respondents). Thus, it was merged with the bundle combining mulching, compost manure, and crop diversification and analysed as one group. Therefore, this study ended up with seven bundles for empirical estimations that included non-adopters.

**Table 2:** Specification of bundles of various ecological farming practices combinations.

Choice	Ecological farming packages	M	C	D	T	P	Frequency	Percentage
1	Non-adoption						56	17.50
2	CDP		✓	✓		✓	49	15.31
3	CD		✓	✓			46	14.38
4	MCD	✓	✓	✓			43	13.44
5	MCDP	✓	✓	✓		✓	36	11.25
6	C		✓				23	7.19
7	MCDTP	✓	✓	✓	✓	✓	19	5.94
8	MC	✓	✓				13	4.06
9	D			✓			6	1.88
10	CDTP		✓	✓	✓	✓	6	1.88
11	CP		✓			✓	5	1.56
12	CT		✓		✓		4	1.25
13	MD	✓		✓			3	0.94
14	MCDT	✓	✓	✓	✓		3	0.94
15	CDT		✓	✓	✓		2	0.63
16	MCP	✓	✓			✓	2	0.63
17	DP			✓		✓	1	0.31
18	MDP	✓		✓		✓	1	0.31
19	MCT	✓	✓		✓		1	0.31
20	CTP		✓		✓	✓	1	0.31
21	MDTP	✓		✓	✓	✓	0	0
22	MCTP	✓	✓		✓	✓	0	0
23	P					✓	0	0
24	M	✓					0	0
25	DT			✓	✓		0	0
26	TP				✓	✓	0	0
27	MT	✓			✓		0	0
28	MDT	✓		✓	✓		0	0
29	MTP	✓			✓	✓	0	0
30	DTP			✓	✓	✓	0	0
31	MP	✓				✓	0	0
Total							320	100

Note: M denotes Mulching, C compost manure, D crop diversification, T minimum tillage, P integrated pest management.

The farmers who did not use any bundles accounted for 17.5 % of the sampled farmers. The bundle comprising compost manure, crop diversification, and integrated pest management was used by 15.31 %. Only 19 households (5.94 %) used all the ecological practices. Adopting a bundle consisting of all ecological practices was low because most farmers were not using minimum tillage on their farms. Out of the 320 sampled households, only 36 were using minimum tillage.

The average farm incomes for various combinations of ecological practices are shown in Table 3. The households that adopted a combination of all five ecological practices earned the highest farm income annually. Overall, the mean farm income for the surveyed households was 210,210.50 Kenya shillings a year.

**Table 3:** The average farm income for various combinations of ecological practices.

Ecological packages	Average farm income in Kenya shillings
MCDTP	442,105.30
MCDP	275,000.00
CDP	250,408.20
MC	189,230.80
CD	175,434.80
MCD	170,892.90
C	155,217.40

### 3.3 Empirical results

#### 3.3.1 Determinants of choice of specific ecological farming packages

The marginal effect estimates for the determinants of the choice of ecological practice packages are shown in Table 4. The results indicate that increasing age by one year decreased the likelihood of adopting mulching, compost manure, and crop diversification by 0.8 %. The negative effect of the age of the household head on the adoption of mulching, compost manure, and crop diversification packages could be because a practice such as mulching is labor-intensive, requiring healthy, risk-bearing, and energetic individuals. Hence, the package may be unattractive to older farmers. This result corroborates that of [44], who found that the farmer's age was negative and statistically significant with all the alternative packages of improved maize varieties and crop diversification.

**Table 4:** Marginal effects estimates of adoption of ecological farmingpackages- MNL selection model.

Variables	MCD dy/dx	CD dy/dx	CDP dy/dx	MCDTP dy/dx<	MCDP dy/dx	C dy/dx
Gender	−0.001	−0.070*	0.007	0.028	0.007	0.025
Age	−0.008***	−0.001	0.004*	0.001	0.001	−0.000
Household size	−0.001	0.038***	−0.003	−0.011	0.015	−0.005
Education	0.005	−0.005	−0.006	0.003	0.000	−0.002
Experience	0.011***	−0.000	−0.004*	−0.004**	0.001	−0.002
Farm size	−0.002	0.039**	−0.068**	0.036***	−0.021	0.007
Drought and floods	−0.039	−0.040	0.097**	−0.156***	−0.063*	0.121**
Membership	0.000	−0.148***	0.051	0.029	0.214***	−0.019
Off-farm activities	0.122***	−0.003	−0.052	−0.101***	0.029	−0.011
Credit	−0.023	0.152***	0.036	0.033	−0.088**	0.028
Slope	0.015	−0.057	0.017	0.025	0.082*	−0.112
Soil erosion	0.023	0.046	0.039	0.026	−0.010	0.077**
Effect of conventional agriculture	0.049	−0.058	0.174**	0.083*	0.007	−0.027
Kamburu ward	0.006	−0.066	−0.042	0.001	0.182***	−0.084**

***, **, * denotes statistical significance at 1 %, 5 % and 10 % level.

The results also revealed that larger households had a 3.80 % probability of choosing the compost manure and crop diversification package. Households with more family members have higher food needs. A package combining compost manure and crop diversification can help households meet their food needs by increasing crop production. Also, they can adopt packages that are relatively labor-intensive, such as the use of compost manure. This result aligns with the findings of [42,45,46], who reported that a strong positive correlation existed between family size and adoption of SAPs.

Adopting a package of mulching, compost manure, and crop diversification was more common in households where the head was involved in off-farm activities. As argued by Refs. [24,42], participation in off-farm activities could earn farmers additional income that enables them to invest in innovative technologies such as SAPs. On the other hand, participating in off-farm activities decreased the probability of choosing a combination of all ecological farming packages. This finding alludes to the fact that participation in off-farm activities diverts time and money away from agricultural activities; hence, fewer resources are available to facilitate agricultural technology adoption. This notion is similar to that of [47], who found that farmers’ engagement in off-farm activities negatively and significantly influenced the adoption of agricultural technologies.

Farmers who had more years of experience were less likely to adopt a comprehensive package. This result suggests that farmers with many years of experience may have established farming practices that work for them and may be hesitant to change their farming methods. The finding corroborates that of [48] that farming experience negatively correlates with adopting improved soybean production technologies. However, the more experienced farmers were, the more likely they were to adopt mulching, crop diversification, and compost manure. To explain this result, farmers with more years of farming experience have gathered enough knowledge and information over the years that allows them to adapt to innovations easily. This finding conforms with that of [49] who found that the farming experience positively influences the adoption of improved agricultural technologies.

Farm size played a vital role in the adoption of ecological packages. Large farms increased the probability of adopting a comprehensive package and another package consisting of composting and crop diversification. This result is compatible with those of [21,44,50], who argue that farmers with larger farm sizes benefit from economies of scale by adopting multiple practices at a time to increase farm production for market and profit maximization. However, having a large farm size decreased the probability of adopting a combination of composting, crop diversification, and IPM. Farmers may find it challenging to implement the package above due to the technical expertise required by IPM practices and the complexity of managing compost manure and diverse crops. Therefore, any additional land is dedicated to other farming methods. Similarly [51], found that farm size had an inverse relationship with using SAPs packages.

Weather-related risks such as drought and floods were key in adopting ecological farming packages. Adopting a package combining compost manure, crop diversification, and IPM and that of compost manure were common among households that experienced yield loss due to drought and floods. Farmers adopt these practices to reduce production losses brought about by droughts and floods. Floods are likely to cause an increase in pests and can also significantly alter the level of plant-available nutrients in the soil. Hence, farmers manage the problems mentioned earlier by applying compost manure and using IPM practices. Also, crop diversification can help manage drought stress by planting crops that can withstand local climatic conditions. Similar to this finding is that of [52], who found that drought conditions positively influenced the adoption of multiple climate-smart agricultural practices. On the other hand, drought and floods decreased the probability of adopting a package consisting of mulching, compost manure, crop diversification, minimum tillage, and IPM. Drought and floods may increase potential crop and income loss risks and uncertainties. Since farmers are rational producers, they tend to prioritize short-term benefits, such as increasing yields and income, over long-term benefits, such as environmental conservation. Therefore, they choose to continue using conventional farming methods as they perceive them to be capable of offering more stable yields and income. Similar to this are the findings of [25] which show that waterlogging and drought stress negatively influence the adoption of sustainable agriculture practice packages.

The results reveal the importance of social capital in adopting ecological packages. The adoption of mulching, compost manure, crop diversification, and IPM packages was higher among households that belonged to a group. Households belonging to a farmer group or organization had exposure to training, information, and credit access. Therefore, farmers who belonged to producer groups could adopt a larger package of ecological practices. Contrary to expectation, group membership was negatively associated with adopting compost manure and crop diversification. Perhaps some of the farmer groups had goals focused more on other group activities, such as welfare and table banking; hence, less information on farming practices was shared. Similarly [49,53], reported a significant and negative relationship between group membership and adopting agricultural technologies.

Credit access had a positive association with the use of a package, including compost manure and crop diversification. Having access to credit enables farmers to cover farm production costs. This result is consistent with [51], who reported that credit access significantly and positively influenced the adoption of climate-smart agricultural technology packages. On the other hand, having access to credit decreased the likelihood of using mulching, compost manure, crop diversification, and IPM. Using larger packages of ecological practices may increase the risks and uncertainties about potential crop failure arising from increased pressure from pests and market uncertainties associated with crop diversification. Therefore, the acquired credit is mainly invested in other production practices or non-farm activities. Similarly [48], found an inverse relationship between access to credit and adopting multiple agricultural technologies.

Adoption of compost manure, crop diversification, and IPM was higher among households that perceived conventional agriculture to have harmful effects. Soil degradation, low food production, high cost of chemical inputs, pesticide residues in crops, and unpredictable weather were significant concerns for farmers in the study. Farmers adopted farming practices that could restore degraded soils while increasing production of healthy food. Compost manure and IPM were used to replace chemical fertilisers and pesticides, while crop diversification was used as a risk mitigation strategy against crop and income loss. The results further indicate that households in the Kamburu ward had a higher probability of adopting larger packages than in the Kirenga ward. This result could be attributed to more NGOs offering extension services and input support to farmers from the Kamburu ward.

#### 3.3.2 Average adoption treatment effects for the ecological farming practices packages

In the second stage, an ordinary least squares (OLS) regression model was used to estimate the effect of each combination of ecological practices on households’ farm income. The self-selection bias from the first stage was handled at this stage. However, for briefness, we did not report the results from the OLS regression. The main focus was to estimate the treatment effects. The treatment effects of adopting a combination of ecological practices on farm income are reported in Table 5. The average treatment effects were estimated by calculating the farm income of households that adopted ecological practices packages (observed scenario) and comparing them with those of households that had not adopted ecological practices (counterfactual scenario). The values of ATTs are presented in Table 5, showing that a household is better off if ATT is positive and worse off if it is negative.

**Table 5:** Treatment effects of adoption of ecological farming packages on farm income using ESR.

Ecological farming practices categories	Treated	Untreated characteristics	Average treatment effects on the treated (ATTs)
MCDTP	12.953	11.863	1.090***
CDP	12.254	11.953	0.301***
MCDP	12.282	12.125	0.157*
C	11.855	12.034	−0.179
CD	11.801	12.004	−0.203**
MCD	11.801	12.140	−0.339***

***, **, * denotes statistical significance at 1 %, 5 % and 10 % level.

The results revealed that a package consisting of all ecological practices (mulching, compost manure, crop diversification, minimum tillage, and IPM) positively impacted farm income. The impact magnitudes of adopting all five ecological farming practices are greater than those of adopting four practices and below. Specifically, adopting all five ecological practices increased farm income by 9.2 %. Farmers who chose to adopt compost manure, crop diversification, and integrated pest management package also had income gains. Further, using a combination of mulching, compost manure, crop diversification, and integrated pest management was statistically significant at the 10 % level. The ATT for the package above was positive, implying that farm income increased for the households that used that package.

However, using packages combining compost manure and crop diversification only and one that consists of mulching, compost manure, and crop diversification reduced farm income by 1.7 % and 2.8 %, respectively. This finding means the adopters of the packages mentioned above would be better off in the counterfactual scenarios (non-adoption). On the other hand, the package, including only composting, was not statistically significant. Nonetheless, the results show a negative ATT, suggesting that households that used only compost manure were worse off.

## 4 Conclusion and recommendations

Many institutions are promoting ecological farming approaches as a viable solution that can deliver sustainable agricultural and food systems that are much needed to achieve food security, protect the environment, and empower societies. This study investigates the factors affecting households’ decisions to adopt a combination of ecological practices and estimates the effects of the adoption on farm income. In contrast to most prior studies conducted in Kenya, which only focus on a single ecological technology, we consider various combinations of the five ecological farming practices (mulching, composting, crop diversification, IPM, and minimum tillage). The results of the adoption effect reveal that the adoption of the package that combines all ecological farming practices provides the highest farm income per year. The comprehensive package replaces chemical inputs with locally available resources, restores degraded soils, and diversifies production risks. Most importantly, our results indicate that farming experience, off-farm activities, farm size, perceptions of the effects of conventional agriculture, and drought and floods significantly influenced the adoption of the comprehensive package.

This study’s findings imply that policymakers can design policy interventions to incentivize farmers to adopt all five ecological practices as a package by capitalizing on the factors significantly influencing the adoption of the package. For instance, the significant role of larger farm sizes in encouraging the adoption of a comprehensive package suggests the need to enforce land use planning laws that discourage further agricultural land fragmentation. Similarly, the correlation between households' perceptions of the effect of conventional agriculture and increased adoption of the comprehensive package underscores the need to establish targeted information campaigns through digital platforms and extension services to educate farmers about the drawbacks of conventional agriculture and the benefits of ecological practices and dispel misconceptions. Furthermore, the results signify the importance of timely weather-related data, including rainfall amounts, time of year, and distribution, to cushion farmers from weather-related risks like drought and floods.

Although this study contributes significantly to the empirical evidence about the effect of ecological farming adoption on farm income in Kenya, it should be noted that the study still has some possible limitations. First, the study only focused on five ecological practices (mulching, composting, crop diversification, IPM, and minimum tillage) that farmers used to reduce production risk, reduce chemical inputs, and manage soil and water. However, farmers adopted other ecological practices based on their local ecological conditions. Second, this study is based on cross-sectional data. Therefore, we did not assess the adoption and effect of packages of ecological practices on farm income over time.

## Funding statement

This work was supported by the funding 10.13039/501100016991African Economic Research Consortium (AERC).

## Disclosure statement

The authors report there are no competing interests to declare.

## Data availability statement

The data presented is available on request from the corresponding author.

## CRediT authorship contribution statement

**Emma Wangari Kamau:** Writing – original draft, Methodology, Investigation, Funding acquisition, Conceptualization. **Raphael Gitau:** Writing – original draft, Methodology, Investigation, Formal analysis, Conceptualization. **Hillary K. Bett:** Writing – original draft, Methodology, Investigation, Formal analysis, Conceptualization.

## Declaration of competing interest

The authors declare that they have no known competing financial interests or personal relationships that could have appeared to influence the work reported in this paper.
